# Supplementary material for: Adherence to behavioral Covid-19 mitigation measures strongly predicts mortality
Source: PLoS One. 2021 Mar 30;16(3):e0249392. doi: 10.1371/journal.pone.0249392 (PMC8009358; doi:10.1371/journal.pone.0249392)
Supplement: Attachment — Submitted filename: Response to Reviewers.docx [file pone.0249392.s002.docx]

**List of changes:**

| **Comment of the Editor/Reviewer** | **Change – page references refer to the revised manuscript** | **Our Comment** |
| --- | --- | --- |
| *Editor* |  |  |
| Thank you for submitting your manuscript to PLOS ONE. After careful consideration, we feel that it has merit but does not fully meet PLOS ONE’s publication criteria as it currently stands. Therefore, we invite you to submit a revised version of the manuscript that addresses the points raised during the review process.  When submitting your revision, we need you to address these additional requirements. | Please, see answers to the reviewers. | Thank you very much for the possibility to submit a revised version of our manuscript. We have considerably revised the original manuscript by addressing each point raised by the reviewer and the additional requirements of the Journal. This remarkably improved our work. We really hope to have covered all the suggestions adequately. |
| 1.) Please ensure that your manuscript meets PLOS ONE's style requirements, including those for file naming. … |  | Our manuscript meets PLOS ONE’s style requirements, including those for file naming. |
| 2.) In your Methods section, please provide additional information about the participant recruitment method and the demographic details of your participants. Please ensure you have provided sufficient details to replicate the analyses such as: a) the recruitment date range (month and year), b) a description of how participants were recruited, and c) descriptions of where participants were recruited and where the research took place.  Moreover, please provide a rationale for the inclusion of the specific 8 countries, and for investigating mortality rates between in the period  June - August 2020. " | *Page 5-6:*  “Data were collected within ten days between May 28 and June 7, 2020 by an independent social marketing and research institute (YouGov, www.yougov.de) through online population-based panel surveys in the national language of the countries. The participants were recruited from the resident population, and were aged 18 years and older. To achieve representativeness, a stratification by age, gender and region was performed. In all countries, participation was compensated by panel-specific tokens that can be converted into vouchers or cash payments. The main research work took place in Germany.”  *Page 7:*  Table 2  *Page 4-5:*  “Building on and extending earlier work on the link between macrosocial factors and mental health [15, 16], we selected the United States, Russia, Poland, Sweden, Germany, France, Spain and the United Kingdom for our study. These countries not only represent different types of societies and health care systems, but also differ in their emphasis on personal freedom, government effectiveness and attitudes to NPIs [15-18]. In most countries, the first cases of Covid-19 were reported in January 2020 (except Poland, which reported first cases in March 2020) and governments and health authorities subsequently advocated behavioral measures to contain the pandemic. With the exception of Sweden, all countries declared total or partial lockdowns in March 2020, which were eased from April or May 2020 (see Table 1). Table 1 shows the times of lockdowns in spring 2020 and the governmental NPIs in the eight investigated countries that were effective in the end of May 2020 and in the beginning of June 2020.”  *Page 5:*  Table 1 | The information you requested is included in the manuscript. Table 2 presents the demographic details of the samples. The information about the participant recruitment method is included in the Method section. We have provided sufficient details to replicate the analyses:  a) The recruitment was conducted within ten days between May 28 and June 7, 2020.  b) and c) Participants were recruited by an independent social marketing and research institute (YouGov, www.yougov.de) through an online population-based panel. We added the URL of the online-page of YouGov in the revised manuscript to enable readers to get more information about this independent social marketing and research institute.  The authors of the present study are located in Germany (see the affiliations). Thus, the work on the research data was conducted in Germany. We included this information in the revised manuscript.  Our research group has previously conducted two cross-national studies on these eight countries due to their different welfare system (please see for detailed explanation Scholten, Velten, & Margraf, 2018; Scholten, Velten, Neher, & Margraf, 2017). Against this background, the present data collection has been considered as a continuation of our previous research. Moreover, the eight countries not only represent different types of societies and health care systems, but also differ in their emphasis on personal freedom, government effectiveness and attitudes to behavioral anti-Covid-19 measures (e.g., Czeisler et al., 2020; Liang et al., 2020). We included this information in the Introduction section of the revised manuscript. Furthermore, following the advice of the reviewer, we included a table with country specific anti-Covid-19 measures in the revised manuscript.  Data on adherence in the eight countries were collected between May 28 and June 7, 2020. Considering that in June 2020 nobody knew how the Covid-19 situation will develop, we did not a priori plan to assess further data after the data collection. However, considering the worldwide high mortality rate caused by Covid-19 in the summer months, it was urgent to understand its predictors to contribute to the pandemic fight. Therefore, we decided posteriori to investigate whether adherence assessed in June can predict the mortality rate. Considering our significant findings, a timeframe of three months is informative for this issue. We hope that you consider this explanation as sufficient.    References:  Czeisler, M. É., Tynan, M. A., Howard, M. E., Honeycutt, S., Fulmer, E. B., Kidder, D. P., . . . Baldwin, G. (2020). Public attitudes, behaviors, and beliefs related to COVID-19, stay-at-home orders, nonessential business closures, and public health guidance—United States, New York City, and Los Angeles, May 5–12, 2020. *Morbidity and Mortality Weekly Report, 69*(24), 751. doi:10.15585/mmwr.mm6924e1  Liang, L.-L., Tseng, C.-H., Ho, H. J., & Wu, C.-Y. (2020). Covid-19 mortality is negatively associated with test number and government effectiveness. *Scientific reports, 10*(1), 1-7. doi:10.1038/s41598-020-68862-x  Scholten, S., Velten, J., & Margraf, J. (2018). Mental  distress and perceived wealth, justice and freedom  across eight countries: The invisible power of the  macrosystem. *PLoS One, 13*(5), e0194642.  doi:10.1371/journal.pone.0194642  Scholten, S., Velten, J., Neher, T., & Margraf, J.  (2017). Wealth, justice and freedom: objective and  subjective measures predicting poor mental health in a  study across eight countries. *SSM-Population Health,*  *3*, 639-648. doi:10.1016/j.ssmph.2017.07.010 |
| 3.)  We note your statement "All required permits and approvals for the data collection in the eight countries were obtained". Please specify the names of the IRBs that approved the study in the 8 countries. | *Page 6:*  “The study was approved by the ethics committee of the Faculty of Psychology of the Ruhr-Universität Bochum (Germany) and pre-registered with AsPredicted.org on May 25, 2020 (https://aspredicted.org/e7a9g.pdf). All required permits and approvals for the data collection in the eight countries were obtained by the independent social marketing and research institute YouGov.” | The study was approved by the ethics committee of the Faculty of Psychology of the Ruhr-Universität Bochum. In addition, the independent social marketing and research institute YouGov that conducted the data collection obtained all required permissions and approvals for the conduction of international data collections. We included this information in the revised manuscript. |
| 4.) We noted in your submission details that a portion of your manuscript may have been presented or published elsewhere.  "The present data set has been used for the preparation of an other manuscript that is submitted to PLOS ONE (PONE-D-20-27521R1), but that paper has another main focus. Editor for the other manuscript is Antonio Scala."  Please clarify whether this publication was peer-reviewed and formally published. If this work was previously peer-reviewed and published, in the cover letter please provide the reason that this work does not constitute dual publication and should be included in the current manuscript. |  | The publication was peer-reviewed and formally published:  Margraf, J., Brailovskaia, J., & Schneider, S. (2020).  Behavioral measures to fight COVID-19: An 8-country  study of perceived usefulness, adherence and their  predictors. *PLoS One, 15*(12), e0243523.  doi:10.1371/journal.pone.0243523  We included the reason that this work does not constitute dual publication and should not be included in the current manuscript in the cover letter. |

| **Comment of the Editor/Reviewer** | **Change – page references refer to the revised manuscript** | **Our Comment** |
| --- | --- | --- |
| *Reviewer 1* |  | Thank you very much for the insightful comments. We have considerably revised the original manuscript by addressing each point raised by you. This remarkably improved our work. We really hope to have covered all the suggestions adequately. |
| Abstract  Keeping distance should read maintaining social or interpersonal distance Benefits questioned by whom? | *Page 2:*  “In the absence of vaccines or causal therapies, behavioral measures such as wearing face masks and maintaining social distance from are central to fighting Covid-19. Yet, their benefits are often questioned by the population and the level of adherence to the measures is variable.” | We apologize the unclear formulation. We reformulated both sentences in the revised manuscript. |
| Syntax and grammatical mistakes evident. |  | Thank you for this advice. The revised manuscript has been proof-read by an English native speaker. |
| Introduction Page 1- Line 56-Rather than dynamics this should state reduce ‘R’ associated with transmission | *Page 3:*  “Early experiences from China pointed to the effectiveness of massive NPIs in shortening the serial interval of SARS-CoV-2 infections over time and in reducing the transmission of the virus [3-5].” | We corrected this formulation in the revised manuscript. |
| Page 1- Line 59- this should be re-written as it suggests the introduction was written in May of 2021 when most countries are currently in lockdown. Authors should state that in May of 2020 there was some easing of government implemented ant-pandemic measures | *Page 3:*  “Since May 2020, some countries have started to ease or lift some of the government implemented anti-pandemic measures.” | We corrected this formulation in the revised manuscript. |
| Syntax and grammatical mistakes evident |  | Thank you for this advice. The revised manuscript has been proof-read by an English native speaker. |
| Method & Results  Is there an explanation for the far higher percentage of individuals aged over 45 across all countries.  Perhaps a table would be useful describing the specific anti-pandemic measures in place at the time of data collection across the participant countries. This is important as the study rests on the idea that adherence is related to morality and therefore the paper might be stronger if the specifics for each country are clearly identified. | *Page 14:*  “Fourth, data of adherence were collected by an independent social marketing and research institute. To achieve representativeness, stratification by age, gender and region was performed. Thereby, the age distribution was quoted and weighted representative of the population from 18 years of age in each country. Thus, only respondents aged 18 and older were considered; respondents under 18 were not taken into account in the age distribution. As a consequence, higher proportions of the samples fall to the older population groups.”  *Page 5:*  Table 1 | Participants were recruited by an independent social marketing and  research institute (YouGov, www.yougov.de) through an online  population-based panel. To achieve representativeness, a  stratification by age, gender and region was performed. Following  the stratification rules of YouGov, the age distribution is quoted and  weighted representative of the population from 18 years of age in  each of the eight countries. Only respondents aged 18 and older are  considered; respondents under 18 years are accordingly not taken  into account in the age distribution. As a consequence, higher  proportions fall to the older population groups. This explains the  higher percentages of individuals aged over 45 across the  investigated countries. We included this information to the  limitations in the revised manuscript.  Following your suggestion, we included a table describing the  specific anti-pandemic measures at the time of data collection across  the participant countries in the revised manuscript. |
| Its of critical importance that the number of deaths from the virus in each of the participating countries is accurate. Therefore, the source of this information should be clearly identified. Do the figures match on WHO statistics? There is a reference (19) provided but what is the validity and accuracy of this data? | *Page 8:*  “Mortality data for June 1, 2020 and August 31, 2020 were taken from published sources that receive data from the Covid-19 Data Repository by the Center for Systems Science and Engineering (CSSE) at Johns Hopkins University (JHU, USA) [27].” | We agree that it is of critical importance that the number of deaths  from the virus of each of the participating countries is accurate.  The number of deaths from the virus in each of the participating  countries is taken from Hasell et al. (2020). This is an online-  page that provides daily updated Covid-19 data from each country  that we investigated. The data come from the Covid-19 Data  Repository by the Center for Systems Science and Engineering  (CSSE) at Johns Hopkins University (JHU). Thus, the data on the  number of deaths are accurate. We included this information in the  revised manuscript.  Reference:  Hasell, J., Mathieu, E., Beltekian, D., Macdonald, B., Giattino, C., Ortiz-Ospina, E., . . . Ritchie, H. (2020). A cross-country database of COVID-19 testing. *Scientific data, 7*(345), 1-7. doi:10.1038/s41597-020-00688-8 |
| There is no breakdown of adherence relative to age. This might be useful to consider. |  | Considering your concern, we calculated the relationship between  the variables age group and adherence for the overall sample (*r* =  .087, *p* < .001) and for each of the eight investigated countries  separately (GE: *r* = .224, p < .001, FR: *r* = .069, *p* < .05, ES: *r* =  .070, *p* < .01, PL: *r* = .118, *p* < .001, RU: *r* = -.001, n.s., SV: *r* =  .080, *p* < .05, UK: *r* = .065, *p* < .01, US: *r* = .071, *p* < .05). In most  of the investigated countries, this relationship is positive, but weak.  This is an interesting issue, but its inclusion in the main manuscript would interrupt the reading flow because the relationship between age and adherence is not the main topic of the present investigation. We hope that you understand this. If you consider this as important, we can include the correlation results as supplementary material. |
| Discussion There really should be a richer discussion of the psychological factors involved in non-adherence other than whether governments were ambivalent about them. | *Page 12-13:*  “In addition to the form and perception of the governmental communication, further factors can impact the individual adherence to the anti-Covid-19 measures. For instance, many people use social media such as Facebook and Twitter as source of Covid-19 information [28]. In contrast to other information sources such as television reports, newspaper reports and official sites of federal government and authorities, content provided on social media is user-generated. Thus, each user can create, modify and share the content [29, 30]. As a consequence, social media often provide a high amount of unfiltered (mis)information [31]. Previous research on earlier extraordinary societal situations (e.g., terrorist attacks, epidemics) [32, 33] and recent research on Covid-19 [34, 35] showed that the consumption of such information can contribute to emotional overload, enhanced stress symptoms, experience of burden and reduction of adherence to urgent measures. To prevent the negative impact of social media use and to increase adherence to the NPIs, a stronger control of the content provided on social platforms by the providers is urgent. In addition, governmental communication should stress the responsibility for the generated online content of each user and the need to verify all information through official sources before sharing.  Moreover, individual cost-benefit considerations can influence the level of adherence to the NPIs. Measures such as social distancing, limited leisure travel, wearing of face masks, frequent washing of hands and fever measuring reduce the risk for infection and thus in the longer-term contribute to the reduction of the pandemic spread and of the mortality rate [11]. However, in the short-term, they can be experienced as inconvenient and restrictive [2]. People who perceive the short-term costs of the measures as higher and more significant than the longer-term benefits tend to low adherence to the NPIs, especially when they rate their own risk for infection as low [13, 36]. Therefore, programs that focus specifically on these individuals and emphasize the longer-term benefits of adherence are required.  A further factor that might impact the adherence to the NPIs is sense of control. Sense of control belongs to important humans needs [37]. People with a low level of sense of control often have enhanced stress and anxiety symptoms. They tend to rumination and maladaptive coping-strategies such as problematic substance use [38]. In a recent study, low sense of control was positively associated with the experience of burden by the Covid-19 situation [39]. Against this background, it can be assumed that people with a low level of sense of control are at risk for low adherence to the NPIs because they do not trust in the efficacy of their own activities and thus are convinced that their behavior cannot contribute to the pandemic fight. Governmental communication should emphasize that the adherence to the anti-Covid-19 measures of each individual is important for the control of the current situation and thus for the pandemic fight.” | Following your advice, we included a richer discussion of the psychological factors that could be involved in non-adherence in the revised manuscript. |
| Accuracy of single item to assess adherence and single time point measure should be addressed | *Page 14:*  “Third, the present study is quasi-experimental, drawing inferences from self-reported adherence assessed at only one measurement time point to country-wide mortality figures, and thus may be subject to participant biases, and can only illuminate correlational relationships, and not causation. Furthermore, adherence was assessed by a single-item measure. Available cross-sectional and longitudinal research reported single-item scales that measure various psychological and behavioral constructs such as risk-taking to have adequate psychometric properties [40-44]. Nevertheless, future studies are recommended to include measures that assess adherence to specific NPIs at several measurement time points to gain a more detailed view of the Covid-19 situation.” | Following your advice, we addressed the accuracy of a single item measure to assess adherence and of a single time point measure in the revised manuscript. |
